# Can pulsed ultrasound increase tissue damage during ischemia? A study of the effects of ultrasound on infarcted and non-infarcted myocardium in anesthetized pigs

**DOI:** 10.1186/1471-2261-5-8

**Published:** 2005-04-15

**Authors:** Göran K Olivecrona, Bjarne Madsen Härdig, Anders Roijer, Mattias Block, Edgars Grins, Hans W Persson, Leif Johansson, Bertil Olsson

**Affiliations:** 1 Department of Cardiology, Lund University, SE-22185 Lund, Sweden; 2 Department of Pathology, Lund University, SE-22100 Lund, Sweden; 3 Departement of Anaesthesiology, Lund University, SE-22100 Lund, Sweden; 4 Electrical Measurements, Lund Institute of Technology, SE-22100 Lund, Sweden

## Abstract

**Background:**

The same mechanisms by which ultrasound enhances thrombolysis are described in connection with non-beneficial effects of ultrasound. The present safety study was therefore designed to explore effects of beneficial ultrasound characteristics on the infarcted and non-infarcted myocardium.

**Methods:**

In an open chest porcine model (n = 17), myocardial infarction was induced by ligating a coronary diagonal branch. Pulsed ultrasound of frequency 1 MHz and intensity 0.1 W/cm^2 ^(I_SATA_) was applied during one hour to both infarcted and non-infarcted myocardial tissue. These ultrasound characteristics are similar to those used in studies of ultrasound enhanced thrombolysis. Using blinded assessment technique, myocardial damage was rated according to histopathological criteria.

**Results:**

Infarcted myocardium exhibited a significant increase in damage score compared to non-infarcted myocardium: 6.2 ± 2.0 vs. 4.3 ± 1.5 (mean ± standard deviation), (p = 0.004). In the infarcted myocardium, ultrasound exposure yielded a further significant increase of damage scores: 8.1 ± 1.7 vs. 6.2 ± 2.0 (p = 0.027).

**Conclusion:**

Our results suggest an instantaneous additive effect on the ischemic damage in myocardial tissue when exposed to ultrasound of stated characteristics. The ultimate damage degree remains to be clarified.

## Background

More then 25 years ago, it was reported that ultrasound (US) may enhance the fibrinolytic process [1]. Throughout the 1980s and 2000s, several in-vitro and in-vivo experiments verified and further explored this effect, using US alone [2] or as a thrombolytic adjuvant [3-13]. The concept has recently been explored in myocardial ischemia [14] and infarction [15] in humans.

The physical properties of US fields that may account for the observed profibrinolytic effects include thermal effects, the cavitation effect and micro-streaming [8,11,16,17]. It is, however, still unclear as to how these contribute to the profibrinolytic mechanism. Although these effects of US are beneficial in US enhanced fibrinolysis, they might be harmful to biological tissue in other circumstances, for instance, in already injured tissue. The mechanisms by which US may be potentially harmful to biological tissue are in fact similar to those described in connection with US enhanced fibrinolysis [18-24].

The possible net benefit of successful US enhanced thrombolysis in the setting of myocardial infarction is the result of an earlier reperfusion, this could be diminished by the possible unfavourable effect of US on the ischemic myocardium. We have therefore undertaken this safety study exploring the effects of exposing ischemic myocardium to US. For this purpose a porcine model was developed in which blinded histopathological examination technique was used.

## Methods

### Transducer calibration and measurements

Before the animal experiments, calibration and measurements of US fields were performed on the two unfocused piezoelectric transducers used (Ceram AB, Lund, Sweden). The transducers had a resonance frequency of 1.0 MHz and diameter 16 mm. The transducers were excited by an electronic system consisting of a function generator (HP 3314A, Hewlett-Packard, Washington, USA) and a RF power amplifier (ENI 240L, ENI, Rochester, New York, USA).

### Electronic scale measurements

Measurements to determine the total radiation force were performed, using an electronic scale (Model UPT-DT-1, OHMIC Instrumental Co, St Michaels, Maryland, USA). US of 1 MHz and a spatial-averaged, temporal-averaged intensity (*I*_SATA_) of 1 W/cm^2 ^was sent as continuous wave and US of 1 MHz and a intensity of 1 W/cm^2 ^(I_SATA_) was sent as pulsed wave (one burst of 100 pulses per millisecond). The continuous US exposure constituted a reference measurement and was only used in the electronic scale measurements.

### Hydrophone measurements

US of 1 MHz and an intensity of 1 W/cm^2 ^(*I*_SATA_) was sent as pulsed wave (one burst of 100 pulses per millisecond). Measurements were performed by scanning with a polyvinylidene fluoride membrane hydrophone, (GEC-Marconi Hydrophone Type Y-34-3598, Calibrated at National Physical Laboratory, Teddington, England) to determine the Mechanical Index (MI), the peak compressional pressure, rarefactional pressure and the maximal spatial peak temporal average Intensity (*I*_SPTA_). The signal was registered on an oscilloscope (Tektronix TDS 360, Tektronix UK, Ltd. Berkshire, United Kingdom).

Measurements were also performed of the distribution of the US field yielded by a comparable transducer as used in the study. Scanning was performed with a 0.5 mm diameter needle hydrophone and amplifier (Precision Acoustic LTD. United Kingdom). From the oscilloscope, digitised signals were transferred into a computer program based on Lab-View software (Department of Electrical Measurements, Lund Institute of Technology, Lund, Sweden). The computer-controlled scanning-system enabled the hydrophone to be translated along three orthogonal axes (X, Y and Z). Scanning was performed over an area of 80 × 30 mm^2 ^in the Y and Z-directions starting close to the transducer surface.

### Temperature measurements

Control measurement was performed of pulsed US exposure effects on temperature rise on non-circulated pig myocardium. One 0.5 mm temperature probe was placed 1.5 cm inside an extracted pig myocardial muscle (3.0 cm thick) with no circulation [25]. The myocardial muscle was then placed in a degassed water bath that was heated to 37°C. The US transducer was placed 1.5 cm perpendicular to the myocardial muscle surface and centred to the temperature probe. Pulsed US exposure started when water bath and muscle reach equivalent temperature. US of 1 MHz and an intensity of 1 W/cm^2 ^(I_SATA_) was sent as pulsed wave (one burst of 100 pulses per millisecond) during one hour. Simultaneous temperature measurement was performed inside the myocardial muscle and in the surrounding water bath once every half-second during the one hour of pulsed US exposure.

### Animal preparation

The study was approved by the Ethical Committee of the University of Lund (approval M246/91). Seventeen 25–30 kg Swedish landscape pigs were used in the study. Anaesthesia was induced with 5–10 ml (25 mg/ml) sodium pentothal (Pentothal Natrium, Abbot Scandinavia AB, Sweden) intravenously (I.V.) before tracheotomy. The pigs were mechanically ventilated (Serviventilator 900 B, Siemens Elema, Sweden). Access to circulation was maintained through one arterial entrance and at least two venous lines. Blood pressure was continuously monitored through the arterial line. Anaesthesia was maintained with ketamine (Ketalar, Parke-Davis, Division of Warener Lambert Nordic AB, Sweden) at a dose of 5 mg/Kg/min I.V., and pancurone (Pavulone, Organon Teknika AB, Sweden) at a dose of 0.3 mg/Kg/min I.V. Following sternotomi, the pericardium was incised and its borders along the incision line sutured to the skin overlying the sternal edges. A large proximal diagonal branch of the left anterior descending artery (LAD) was ligated to induce myocardial infarction.

### Transducer and US exposure

Following 1 hour of coronary ligation, the US transducers were applied. Each of the two transducers was fixed to a universal joint attached to a small steel pipe on a stand. The transducers were thus in a fixed position, and placed approximately 1.5 cm from the epicardium.

**Figure 1:**
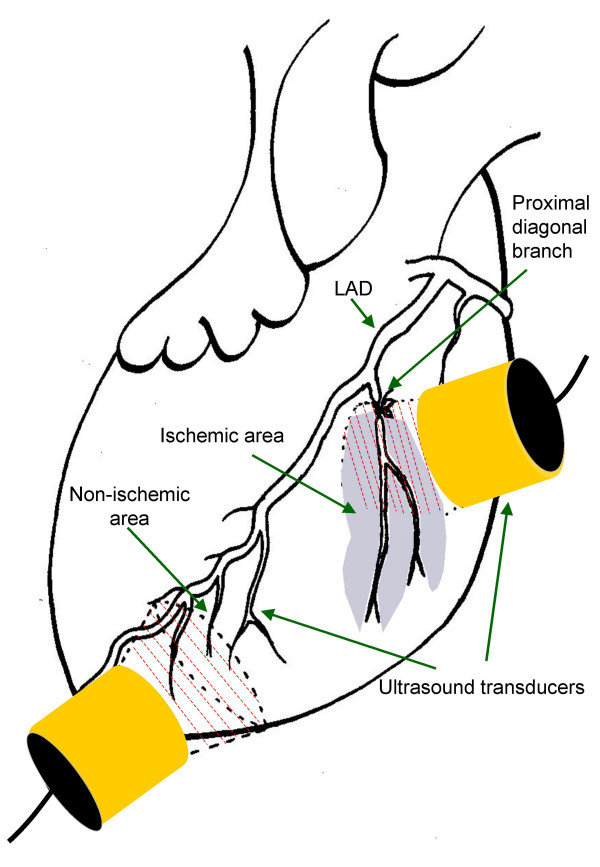
**Experimental setup**. Schematic depiction of the open chest porcine model with the ligated diagonal branch of the left anterior descending artery. The shading indicates the extent of ischemic tissue. Ultrasound transducers are applied over ischemic as well as non-ischemic tissue.

The transducer at the non-infarcted myocardium was placed to radiate part of the anterior/apical free wall of the left ventricle, while the transducer radiating part of the infarcted myocardium was placed in the mid/basal region of the anterior portion of left ventricle, corresponding to the myocardial region perfused by the ligated large diagonal branch of the LAD (Figure 1). US gel (Clinical, Diagramm Halbach AG, Germany) was then applied to the entire anterior portion of the heart to ensure adequate sound wave transmission to the epicardium. Due to loss of gel during the procedure, additional gel was deposited repeatedly during the one hour transducer and pulsed US exposure period.

The US radiation applied over the myocardial areas was, pulsed US of frequency 1 MHz and intensity of 1 W/cm^2^. Each millisecond, a burst of one hundred cycles was sent, equivalent to a duty cycle of 10% and a resulting intensity of 0.1 W/cm^2 ^(*I*_SATA_).

The experiment was designed to illustrate possible injury effects of pulsed US exposure, mechanical handling of the hearts and application of transducers alone. The altogether 68 tissue samples from the 17 pig hearts were thus used as follows:

A) 17 samples of non-infarcted myocardium without any exposure.

B) 4 samples of non-infarcted myocardium exposed to transducer alone.

C) 13 samples of non-infarcted myocardium exposed to pulsed US.

D) 17 samples of infarcted myocardium without any exposure.

E) 8 samples of infarcted myocardium exposed to transducer alone.

F) 9 samples of infarcted myocardium exposed to pulsed US.

### Tissue preparation and histopathological evaluation

After one hour of applied pulsed US radiation or transducer exposure, epicardial sutures were placed at two locations under the transducers, to indicate the diameter of the exposed area and indicate areas were tissue samples should be removed. The pigs were then immediately given 10 ml of potassium (2 mmol/ml, Addex-Kalium™, Pharmacia & Upjohn AB, Sweden) intravenously to induce ventricular fibrillation, which occurred momentarily following administration. The great vessels were then clamped and cut by scalpel, while the veins were ligated and cut by scissors. The entire heart was then extracted intact followed by immediate removal of tissue samples. During the procedure, care was taken to minimize traumatic handling of the heart. Transmural samples, 5–10 mm in diameter, were then carefully removed with a scalpel. Following excision, the tissue samples were prepared for histopathological evaluation. From each sample, three slides were cut from the formalin fixed and paraffin embedded blocks and stained with Hematoxylin-Eosin (Van Gieson, and Phosphor Tungistic Acid Hematoxylin, PTAH) respectively. The samples were then examined by routine light microscopy in 10×, 20× and 40× enlargement. An experienced pathologist examined all slides in a blinded fashion unaware of whether the tissue samples were from infarcted, or non-infarcted tissue, or if it had, or had not been exposed to US. In the microscopic evaluation only the common signs of tissue damage, seen during the early phase of myocardial infarction, were observed [26].

The parameters evaluated for damage score were as follows:

1) Eosinophilic changes in the myocyte, (Ischemia)

2) Reduction and/or loss of cross striation, (Loss of striation)

3) Coagulation necrosis, (Necrosis)

4) Infiltration of polymorphonuclear cells, (PMN)

All parameters were scored from 0 to 3, where 0 indicated no damage, 1 indicated minimal change, 2 intermediate changes and 3 extensive changes. Analysis was made of the damage score for each of the parameters (Ischemia, Loss of striation, Necrosis and PMN) and the total sum of damage scores (TDS) of the four parameters. Thus, the TDS had a minimum possible total damage score of 0 and a maximum possible total damage score of 12.

### Statistical analysis

The TDS of all groups were analysed to estimate the normal distribution by Chi-square test for goodness of fit. The statistical analysis compared the TDS scores in two ways: by paired Students t-test for the comparison of effects in individual animals and Student t-test for group comparison. A p value of less than 0.05 was considered statistically significant.

## Results

### Transducer calibration and measurements

#### Electronic scale measurements

In the balance measurements, using continuous wave US, the US power supply was set to yield an acoustic power value of 2.0 W, corresponding to an intensity of 1 W/cm^2 ^(*I*_SATA_) adjusted to the area of the transducers. Using pulsed wave (10% duty cycle) US at the same power supply the intensity was 0.19 W, which correspond to an intensity of 0.1 W/cm^2 ^(*I*_SATA_).

#### Hydrophone measurements

**Figure 2:**
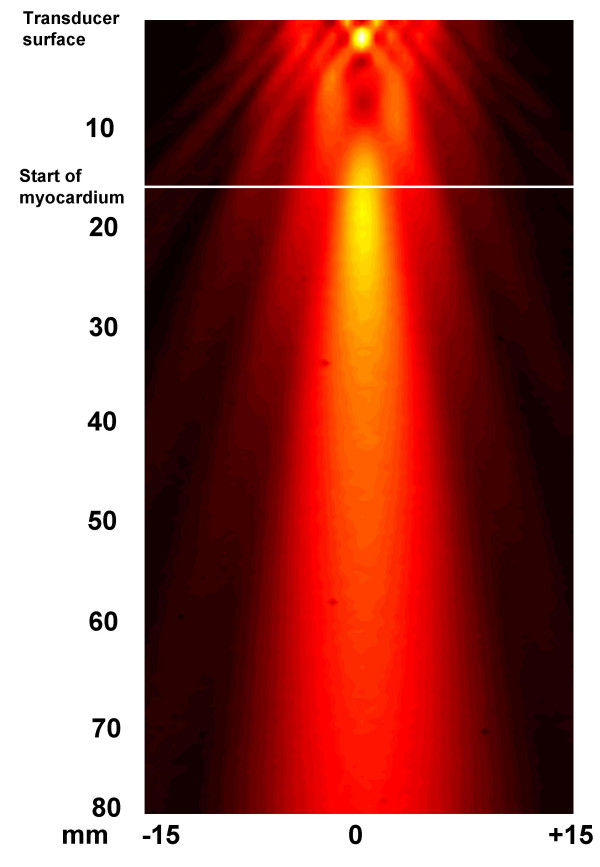
**Ultrasound field distribution**. The field distribution for the transducers is shown, but no exact values of intensity was measured. Scanning was performed over an area of 80 × 30 mm^2 ^in the Y and Z-directions starting close to the transducer surface. The transducer surface and the start of myocardium is mark.

In the membrane hydrophone measurements the *I*_SPTA _was measured to 460 mW/cm^2 ^and the MI was calculated [27] to be 0.41 at a distance of 3.0 – 3.5 cm from the surface of the transducer. At the same distance the peak compressional pressure was measured to a value of 0.41 Mega Pascal (MPa) and the peak rarefactional pressure was 0.41 MPa. The needle hydrophone measurement was performed in degassed water to explore field distribution for the used transducers, the distribution was determined but exact values of intensity were not measured (Figure 2).

#### Temperature measurements

During the first 20 min of pulsed US exposure to the extracted myocardial muscle an increasing temperature difference was seen between the non-circulated pig myocardium and the surrounding water bath. After 20 min of exposure the temperature reached a steady state difference of 0.5°C (Table 1).

**Table 1:** Temperature measurements The temperature increase for non-circulated pig myocardium during pulsed ultrasound exposure. Temperature was measured every 0.5-second during 1 hour and 27 min. Temperature at different time interval is presented as mean ± standard deviation °C. Differences in temperature between the exposed myocardium and surrounding water bath are also shown.

	**Inside water bath**	**Inside heart muscle**	**Difference degrees C**
0.5 sec/intervals	**Mean ± SD**	**Mean ± SD**	
5 min steady state	36.8 ± 0.12	36.8 ± 0.03	0.0
During first 2 min US	36.7 ± 0.12	36.9 ± 0.05	0.2
During first 5 min US	36.8 ± 0.12	37.0 ± 0.07	0.2
During first 10 min US	36.8 ± 0.12	37.1 ± 0.08	0.3
10–20 min US	36.7 ± 0.11	37.2 ± 0.03	0.4
20–40 min US	36.7 ± 0.10	37.2 ± 0.03	0.5
40–60 min US	36.7 ± 0.10	37.1 ± 0.03	0.5
20 min after US termination (2 min)	36.6 ± 0.12	36.7 ± 0.03	0.1

### Physiologic monitoring

All animals were stable in circulation during the experiments and were under supervision of both anaesthesia and cardiology expertise during the whole experiment period. An estimation of the infarcted area is shown in figure 1. No exact measurement of the size of infarcted area was however performed.

### Histopathological evaluation

**Figure 3:**
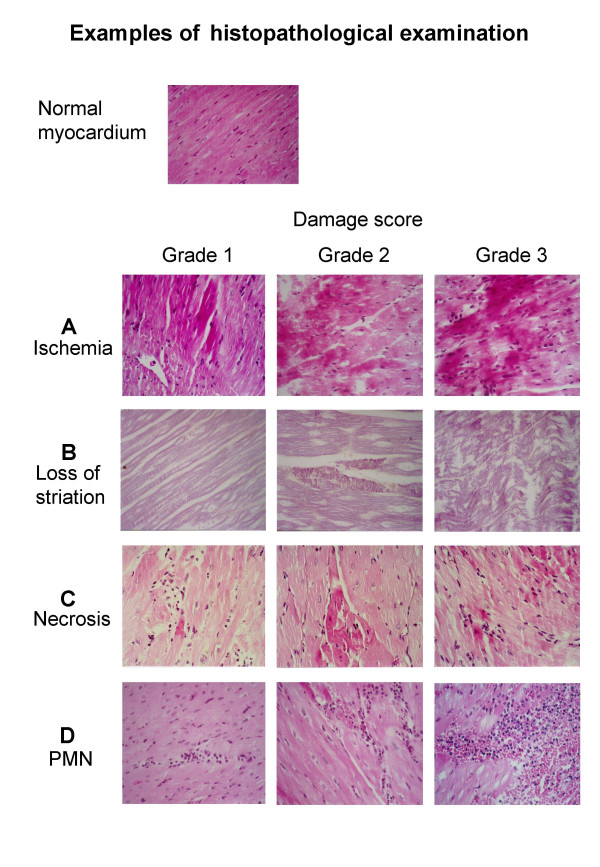
**Examples of histopathological indicators**. Examples of the various histopathological indicators of tissue damage at different damage scores: A = Eosinophilic changes in the myocyte (Ischemia), B = Reduction and/or loss of cross striation (Loss of striation), C = Coagulation necrosis (Necrosis), D = Infiltration of poly-morphonuclear cells (PMN). The estimated degree of damage in each sample is graded in a 0–3 scale. For further information see text.

**Figure 4:**
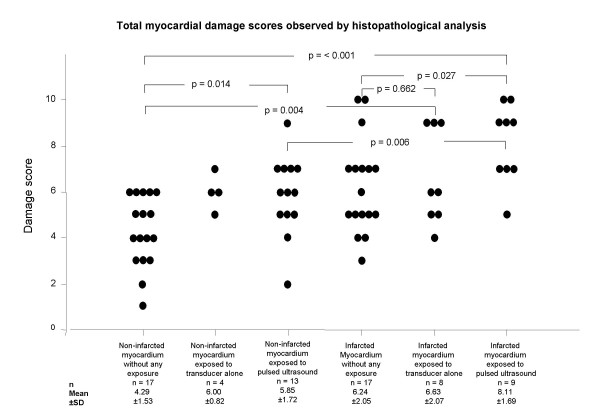
**Individual total damage score and statistical analysis**. The dot-plot shows the total damage scores obtained in 64 individual tissue samples from the 6 different myocardial areas. Statistical comparisons of the total damage scores (TDS) were performed using the Students t-test for group comparison. A p value of less than 0.05 was considered statistically significant.

Examples of the different score grades are shown in figure 3. Signs of myocardial damage were noted already in all 17 perfused tissue specimen untouched by an US transducer, TDS being 4.3 ± 1.5 (mean ± standard deviation). In comparison, the infarcted tissue, untouched by any US transducer, had a significantly higher damage score, irrespective if comparison used the paired difference technique (p < 0.001) or Student t-test for group comparison, 6.2 ± 2.0 vs. 4.3 ± 1.5 (p = 0.004). There was a further significant augmentation of the tissue injury in the infarcted myocardium exposed to US, also evidenced by paired difference (p = 0.026) and Student t-test for group comparison 8.1 ± 1.7 vs. 6.2 ± 2.0 (p = 0.027). The individual damage scores and statistical analyses for all groups are shown in figure 4.

### Control experiments

Following US exposure of non-infarcted myocardium, there was a significant increase in TDS, from 4.3 ± 1.5 to 5.8 ± 1.7 (p = 0.015, Student t-test for group comparison).

The application of transducer exposure alone did not significantly affect the damage caused by myocardial infarction, evidenced by the TDS 6.2 ± 2.0 vs. 6.6 ± 2.1 (p = 0.662, Student t-test for group comparison and p = 0.244 if paired Student t-test was used).

## Discussion

The theoretical possibility of exaggeration of myocardial damage in non-perfused tissue following pulsed US exposure prompted the present analysis of the effect of US on the ischemic myocardium. We chose an open chest porcine model with an induced myocardial infarction and applied US to test our hypothesis that lengthy US exposure on ischemic myocardial tissues *could potentially *be harmful, even when used within limits hitherto considered safe in cardiac exposures [28,29]

The histopathological diagnosis of tissue damage during the first hour of coronary artery occlusion is based on subtle findings. The assessment of cardiac damage used is therefore not specific as a in a fully developed infarction. Furthermore, we found a notable amount of damage already in the samples from the non-infarcted myocardium that not were exposed to transducer or US, a finding which could be explained by stress-induced damage [30-32] as well as mechanical handling of the heart during removal. The genuine effect of the 2-hour long lasting ischemia shows however a significant increase in the total damage score by 45 %. US exposure of the infarcted myocardium resulted in a significant further increase of the total damage score by another 30 %.

### Possible mechanisms of ultrasonic injuries

High Intensity US causes damage in biological tissues through three mechanisms; thermal, mechanical and cavitational injuries [18-24]. The physical properties of US fields which may account for the profibrinolytic effects observed are thermal effects, the cavitation effect and micro-streaming [8,11,16,17]. As earlier stated, the US-induced damage in biological tissues may develop by the same mechanisms that enhance fibrinolysis.

Heat produced in perfused tissues exposed to US within safe levels would under ordinary circumstances mainly be lost to the circulation. In ischemic tissues, the reduced circulation may thus decrease the heat-loss ability during US exposure resulting in undesirable heating and creating a thermal injury [22]. However, in the present study, temperature measurements in the non-circulated myocardial tissue illustrated only a small increase (0.5°C), well within in the limits of now used safety rules.

High frequency US exposure of circulated tissue has been shown to induce a mild increase in interstitial oedema, an accumulation of polymorphonuclear cells [33], oxidative stress in endothelial cells [34] and increased cell lysis and apoptosis in human myelomonocytic leukaemia cells [35]. It has been hypothesized that the effects may be caused by radiation force [35] and other non-thermal effects [34]. US at similar exposure settings as used in the present study have been shown to produce necrotic and cellular damage in epidermis layers in goldfish [25]. Increasing sonication duration resulted in a progressively greater damage score. Damage was also shown to gradually propagate inwards the cell layers with increased sonication duration. It was concluded that the damage produced was caused by cavitational injury [25]. Anatomical structural differences and the age of the animal have also been shown to affect the sensitivity for US exposure [21], although the role of the age dependence remains unclear[24].

In conclusion, available data are unable to disclose the true mechanism of the myocardial injury induced by pulsed US. Finally, the development of ischemic myocardial damage over time is evidenced by different and unspecific indicators of damage, some being reversible [36]. It is therefore not possible to estimate how the myocardial tissue in the present study would be affected after a fully developed infarction.

### Relevance of study results

Multiple studies of the beneficial effects of US used to enhance thrombolysis have been conducted in different animal models, both in the venous and arterial systems [7,8,12,33,37-45]. The progress in the area has also reached humans in the clinical setting. Thus, a prospective controlled study of US augmentation of thrombolysis in myocardial ischemia failed to verify a beneficial effect [14]. In fact, the number of ischemic complications increased following US exposure. In contrast, no undesirable effects were however noticed in 25 patients with myocardial infarction, who received traditional thrombolytic treatment with adjunctive US exposure [15]. Interestingly, early and complete arterial recanalisation was noted in selected patients with stroke, who received thrombolytic treatment and whose cerebral arterial blood flow was followed with transcranial Doppler technique [46].

The result of our study clearly obviates the importance of evaluating the potentially non-beneficial effects of US aiming at enhancement of thrombolysis. In the setting of US exposure during cerebral ischemia, and at US energy levels verified to be beneficial in experimental studies of thrombolysis [7], no detectable additive damage was however verified following exposure of pulsed US [47].

### Study limitations

Two control experiments were carried out. Firstly, we verified that fixation of the transducer and application of US gel close to the infarcted myocardium did not significantly affect the TDS. Secondly, we explored the effect of US on perfused myocardium. Interestingly there were significant signs of myocardial damage also in this presumed healthy tissue following US exposure.

The sensitivity for stress-induced damage in the pig limits this study considerably and makes the interpretation of data more difficult. Still, the total damage score after US exposure significantly exceed damage scores in the unexposed infarcted tissue samples.

No threshold measurements were performed, however the total US energy we used is in the order of 10 times higher than required to enhance the thrombolysis in-vitro[6]. Neither was the temperature monitored in the areas during exposure to US. The low number of samples (n = 4) in the group of non-infarcted myocardium exposed to transducer alone is the result of protocol misinterpretation, unfortunately necessitating the exclusion of these data from the statistical comparison.

## Conclusion

Lengthy pulsed US exposure of low intensity exerting beneficial pro-fibrinolytic effects in the setting of thrombolysis may increase instantaneous myocardial damage.

## List of abbreviations used

US: Ultrasound

*I*_SATA_: Spatial-Averaged, Temporal-Averaged Intensity

MI: Mechanical Index

*I*_SPTA_: Spatial-Peak Temporal Average Intensity

LAD: Left anterior descending artery

TDS: Total damage score

## Competing interests

The author(s) declare that they have no competing interests.

## Authors' contributions

GO designed the investigation, did the surgical and ultrasound experiments, performed the statistical analysis and interpretation of the results, as well as the preparation of the manuscript.

BMH performed the statistical analysis and interpretation of the results, as well as the preparation of the manuscript.

AR was involved in the design of the investigation and interpretation of the results and approved the final manuscript.

MB was involved in the histopathological evaluation and interpretation of the results and approved the final manuscript.

EG did the surgical and ultrasound experiments and approved the final manuscript.

HWP was involved in the designed the investigation and interpretation of the results, as well as the preparation of the manuscript.

LJ was involved in the histopathological evaluation and interpretation of the results and approved the final manuscript.

SBO supervised and designed the investigation as well as participated in the preparation of the manuscript.

All authors read and approved the final manuscript.

## Pre-publication history

The pre-publication history for this paper can be accessed here:


